# Do We Know if You Drive? a Quality Improvement Project Improving Compliance With DVLA Guidelines

**DOI:** 10.1192/bjo.2022.318

**Published:** 2022-06-20

**Authors:** Mia McDade-Kumar, Zainab Bashir, Humaira Aziz, Kealy Hughes, Sara Ormerod

**Affiliations:** 1 University Hospitals Birmingham NHS Foundation Trust, Birmingham, United Kingdom; 2 Birmingham and Solihull Mental Health NHS Foundation Trust, Birmingham, United Kingdom

## Abstract

**Aims:**

Background: The Driving & Vehicle Licensing Agency (DVLA) states: “Doctors and other healthcare professionals should: advise individuals on the impact of their medical condition for safe driving ability and also advise the individual on their legal requirement to notify DVLA of any relevant condition”. Within mental health, the guidance states that depression or anxiety associated with “Significant memory or concentration problems, agitation, behavioural disturbance or suicidal thoughts” must be reported to the DVLA. Aims: Identify whether information was collected on driving status of patients presenting with depression/anxiety and self harm. Identify whether accurate advice was provided and documented. Implement changes that would improve compliance with guidance.

**Methods:**

We reviewed notes to collect baseline data for 3 weeks prior to commencing interventions, then weekly for 2 months from November 2021. Cases were defined as: those presenting to Liaison Psychiatry (LP) with an act of self-harm either on antidepressants or with a confirmed diagnosis of depression/anxiety on their record. Each week, the notes of 10 cases were reviewed for evidence of documentation of driving status and advice regarding DVLA guidelines.

**Weekly Interventions:**

Week 1: Email communication to team highlighting the guidance, responsibilities and where to document.

Week 2: Driving status discussed in handover daily to increase awareness and identify/address concerns.

Week 3: Repeat email to team.

Week 4: DVLA guidance posters placed in LP office.

Week 12: Teaching session by Occupational therapist from regional driving assessment centre.

**Results:**

Data were analysed for 90 patients over 9 weeks. 52% were female, and average age was 33 years. Relevant documentation was only made on week 1 (10%) and week 4 (20%). No documentation of either driving status or advice given were made in any of the other weeks analysed.

**Conclusion:**

Achieving compliance with guidance was difficult. Email communication was the most effective intervention. A group discussion to identify drivers of poor compliance found that clinicians failed to ask as the questions were not routine practice, and some voiced concerns about the potential implications of advice (worsening therapeutic relationship or increasing social isolation/implications for employment). Future plans include adding a prompt about driving on the electronic risk assessment, and specific training in the staff induction.

